# Insulin Resistance Triggers Atherosclerosis: Caveolin 1 Cooperates with PKCzeta to Block Insulin Signaling in Vascular Endothelial Cells

**DOI:** 10.1007/s10557-023-07477-6

**Published:** 2023-06-08

**Authors:** Jingjing Tan, Xiaoguang Li, Ning Dou

**Affiliations:** 1 Department of Anesthesiology and Perioperative Medicine, Shanghai Fourth People’s Hospital Affiliated to Tongji University School of Medicine, Shanghai, 200081 China; 2 Department of Thyroid Breast and Vascular Surgery, Shanghai Fourth People’s Hospital Affiliated to Tongji University School of Medicine, 1279 Sanmen Road, Hongkou District, Shanghai, 200081 China; 3 Department of Vascular and Endovascular Surgery, Changzheng Hospital Affiliated to the Naval Medical University, Shanghai, 200003 China

**Keywords:** Insulin resistance, Caveolin 1, PKCzeta, Insulin signaling pathway, Endothelial injury

## Abstract

**Objective:**

To date, therapies for endothelial dysfunction have primarily focused on ameliorating identified atherosclerosis (AS) risk factors rather than explicitly addressing endothelium-based mechanism. An in-depth exploration of the pathological mechanisms of endothelial injury was performed herein.

**Methods:**

Aortic caveolin 1 (Cav1) knockdown was achieved in mice using lentivirus, and AS was induced using a high-fat diet. Mouse body weight, blood glucose, insulin, lipid parameters, aortic plaque, endothelial injury, vascular nitric oxide synthase (eNOS), injury marker, and oxidative stress were examined. The effect of Cav1 knockdown on the content of PKCzeta and PI3K/Akt/eNOS pathway–related protein levels, as well as PKCzeta binding to Akt, was studied. ZIP, a PKCzeta inhibitor, was utilized to treat HUVECs in vitro, and the effect of ZIP on cell viability, inflammatory response, oxidative stress, and Akt activation was evaluated.

**Results:**

Cav1 knockdown had no significant effect on body weight or blood glucose in mice over an 8-week period, whereas drastically reduced insulin, lipid parameters, endothelial damage, E-selectin, and oxidative stress and elevated eNOS levels. Moreover, Cav1 knockdown triggered decreased PKCzeta enrichment and the activation of the PI3K/Akt/eNOS pathway. PKCzeta has a positive effect on cells without being coupled by Cav1, and ZIP had no marked influence on PKCzeta-Akt binding following Cav1/PKCzeta coupling.

**Conclusion:**

Cav1/PKCzeta coupling antagonizes the activation of PI3K on Akt, leading to eNOS dysfunction, insulin resistance, and endothelial cell damage.

## Introduction

The accumulation of hepatic glucose production in response to insulin resistance (IR) and impaired glucagon signaling inhibition is a major contributor to type 2 diabetes and its complications [1, 2]. Defects in insulin signaling, a major feature of IR in obesity [3], disable pathways that normally inhibit hepatic glucose production; the resulting systemic hyperinsulinemia overstimulates hepatic lipid synthesis and storage [4]. In addition to the metabolic derangements of type 2 diabetes, patients have a 2- to 4-fold increased lifetime risk of cardiovascular disease [5], owing largely atherogenic dyslipidemia induced by deranged hepatic lipid metabolism [6]. The endothelium is located on the inner surface of blood vessels and lymphatic vessels [7]. It can sense the chemical stimulation of cytokines in the blood and regulate vasomotor, inflammatory response, and coagulation activation [8, 9]. When IR occurs, the phosphorylation pathway of phosphatidylinositol kinase/protein kinase B (PI3K/AKT) is drastically blocked [10], and endothelial cells become dysfunctional due to the lack of endothelial nitric oxide synthase (eNOS), thereby promoting the occurrence of atherosclerosis (AS) [11–13]. IR-mediated inflammatory responses and oxidative stress jointly promote endothelial cell damage, which is an important pathological basis for the initiation of AS [14].

As a state of cell membrane invagination, plasma membrane microvesicles, also referred as caveolae, play an indispensable role in the physiology or pathology of various cells, such as cell proliferation, apoptosis, differentiation, angiogenesis, and migrate [15, 16]. Caveolin 1 (Cav1) is an essential protein component of caveolins that is involved in caveolae stability, intercellular material transport and signal transduction, endocytosis, and mitochondrial function control [17]. In addition to playing an important regulatory role in inflammation, Cav1 is a key molecule that regulates the insulin signaling pathway and affects IR [18–20]. Its mechanism is primarily through PKCzeta adsorption and activation of PKCzeta-PKB/Akt coupling, inducing insulin receptor substrate (IRS)/PI3k to activate PKCzeta and PKB/Akt signaling, and its downstream normal glucose transport and glycogen synthesis [21]. Although vascular endothelial cells do not have biological functions such as glycogen synthesis, glucose transport–related mechanisms do exist in endothelial cells [22]. PI3k/Akt is the upstream key signal that regulates the production of eNOS [23, 24]. Hence, combining the aforementioned mechanisms, we hypothesized that Cav1/PKCzeta may antagonize the activation of IRS1/PI3k on Akt, leading to eNOS dysfunction and endothelial cell damage.

Endothelial dysfunction appears to be a reversible process [25]. Nevertheless, to date, therapies for endothelial dysfunction have primarily focused on ameliorating identified AS risk factors rather than explicitly addressing endothelium-based mechanism [26]. As a consequence, a thorough exploration of the pathological mechanisms of endothelial injury will facilitate the development of therapeutic strategies targeting these pathways. Drugs that act on endothelial cells in AS-prone areas to reprogram the expression of their protective phenotype would be beneficial in slowing the progression of atherosclerotic lesions.

## Methods and Materials

### Rodent Modeling

Forty male C57BL/6J mice (aged 8 weeks, 15–20 g; GemPharmatech, Nanjing) were raised in the vivarium with a 12-h light/dark cycle and ad libitum access to food and water. The ambient temperature was controlled at 18~26°C and the humidity was ~55%. The mice were randomly divided into four groups: control, AS model, Sh-NC + model, and Sh-Cav1 + model groups. The mouse tail vein was congested by wiping 75% alcohol, and 100 μL of Cav1 lentivirus or control lentivirus (Hlkbio, Wuhan) was injected to infect the aorta. High-fat diet (HFD) induced AS in mice, and the body weights of the mice were recorded. Thereafter, 8 weeks later, mice were euthanized and aortic tissue was collected.

### Cell Culture and Handling

Human umbilical vein endothelial cells (HUVECs; ATCC) were cultured in an incubator (37°C, 95% air, and 5% CO_2_). Dulbecco’s modified eagle medium (DMEM) with 10% inactivated calf serum was applied for culture. Cells were transfected with shRNAs to knock down Cav1. HUVECs were treated with ox-LDL (Yeasen, Shanghai) to induce oxidative stress, followed by ZIP (PKCzeta inhibitor, 1 μM; ab120993, Abcam) treatment for 40 min [27].

### Blood Glucose and Insulin Testing

Tail vein puncture blood of mice induced by high-fat diet for 8 weeks was used as samples to detect blood glucose and insulin levels. Blood glucose was immediately measured using a glucometer. The remaining blood samples were centrifuged at 5000 rpm (4°C, 15 min), and the supernatant was harvested and stored at −80°C, and serum insulin levels were measured by ELISA. Homeostasis model assessment-IR (HOMA-IR) index = blood glucose × insulin / 22.5.

### Blood Lipid Parameters

Triglyceride (TG), total cholesterol (TC), high-density lipoprotein (HDL-C), and low-density lipoprotein (LDL-C) in serum of mice were detected using a biochemical analyzer.

### Oil Red O Staining

Frozen sections of aorta were stained with Oil Red O (Solarbio, Beijing) to assess the lipid deposition. Sections were rinsed with 60% isopropanol, stained with Oil Red O for 10 min, differentiated with 60% isopropanol, and washed with water for 1–2 min. After counterstaining with hematoxylin for 3 min, microscopic examination (Olympus) was performed.

### H&E Staining

Frozen sections of aorta were stained with hematoxylin solution for 5 min, differentiated with 1% hydrochloric acid alcohol for 2 s, and then stained with eosin for 2 min at room temperature. Sections were dehydrated with gradient alcohol and became transparent using xylene. Specimens were observed under a microscope.

### Immunofluorescence (IF)

The deparaffinized aortic slices were permeabilized in 0.1% Triton x-100 and antigen retrieved. After serum blocking, the aorta tissue sections were incubated with primary antibodies against eNOS (Servicebio, Wuhan, China) or E-selectin (Proteintech, Wuhan, China) and CD34 (Invitrogen) overnight at 4°C, followed by FITC anti-rabbit (Proteintech) and FRITC anti-mouse secondary antibodies (Abcam). Slides were then counterstained with DAPI and results were examined with a fluorescence microscope.

### Indicators of Oxidative Stress

Aorta tissues and HUVECs were lysed and then centrifuged to obtain the supernatant. Following the protein concentration determination with a Nano-300, the supernatant was regarded as sample for the measurement of ROS, MDA, and GSH levels using commercial kits (Beyotime, Shanghai). The values were calculated according to the absorbance obtained from the microplate reader (MD).

### Western Blotting

Proteins were harvested from the tissue homogenate and HUVEC lysate, quantified by Nano-300 and denatured by boiling. After the separation and stacking gels were set up, samples were added to lanes, and electrophoresis was performed to separate the proteins. PVDF membranes (Roche) with blots were obtained by electrotransfer system. The membranes were blocked in skimmed milk and hybridized with primary antibodies (against Cav1, PKCzeta, and PI3k/Akt/NOS pathway–related proteins) and HRP-conjugated antibody (Abcam). Blots were visualized with the ECL reagent (Millipore) and gray values were analyzed with ImageJ software.

### Co-IP

According to the same operation as above, tissue homogenate and cell lysate were obtained. A total of 2.5 μg of PKCzeta or IgG antibody (Abcam) was added to 500 μg of lysate along with 10 μL of protein A+G magnetic beads (GenScript, Nanjing). The whole system was then swirled gently for 2 h to ensure adequate contact. Prior to routine western blot analysis, the supernatant was removed magnetically and the beads were boiled with SDS sample buffer at 95°C for 5 min.

### GSH Pull-Down

This assay was performed using the GST Pull-down Kit (K0077, Dia-an, Wuhan, China) according to the operating instruction. Purified GST-tagged PKCzeta protein (Proteintech Group) was used as a bait protein for binding to tissue or cell extracts. The bait protein was added to the prewashed gel and incubated on a shaker for 3 h. The extracts were added to the spin column and allowed to bind for 4 h. Protein complexes were washed 4 times in buffer, dissociated by boiling in loading buffer prior to western blot analysis.

### CCK8

HUVECs were treated with ox-LDL and ZIP as abovementioned, and then incubated with WST-8 reagent (GlpBio) for 2 h. The absorbance (450 nm) was measured with a microplate reader.

### ELISA

The supernatant of the HUVECs was centrifuged at 500 × g at 4°C for 5 min and then collected. The levels of IL-6, IL-1beta, and TNF-alpha were measured with the corresponding ELISA kits (X-Y Biotechnology, Shanghai). The absorbance (450 nm) was recorded with a microplate reader.

### Statistics Analysis

Data were presented and analyzed in the form of mean ± standard deviation in Prism 8.0. The Shapiro-Wilk test confirmed that the data were normally distributed, and differences were analyzed by one-way or two-way ANOVA and Tukey’s test. *P*<0.05 means significance.

## Results

### Cav1 on AS Symptoms

**Fig. 1:**
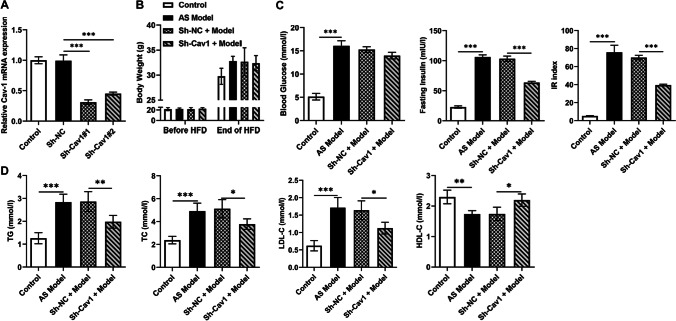
Cav1 on AS symptoms in mice. **A** The knockdown efficiency of lentivirus on Cav1 was evaluated by RT-qPCR. **B** The mouse body weights were recorded over an 8-week period. **C** Blood glucose and insulin were measured and HOMA-IR index was calculated. **D** Triglyceride (TG), total cholesterol (TC), high-density lipoprotein (HDL-C), and low-density lipoprotein (LDL-C) levels in the serum were detected using a biochemical analyzer. ^*^P < 0.05, ^**^P < 0.01, ^***^P < 0.001

The knockdown efficiency of lentivirus on Cav1 was evaluated by RT-qPCR, and the result in the sh-Cav1#1 group was better than that in the #2 group; thus, sh-Cav1#1 was used in subsequent assays (Fig. 1A). Before and after 8 weeks of HFD induction, the body weights of the 4 groups of mice were recorded. The average body weight of mice in the normal group ranged from 21.2 to 29.5 g; the AS model group was from 21.4 to 32.4 g; the Sh-NC + Model group was from 21.3 to 32.1 g; and the Sh-Cav1 + Model group was from 20.9 to 30.8 g. Although there was a slight difference in body weight between the groups, the difference was not significant (Fig. 1B). However, the blood glucose, insulin, and calculated HOMA-IR index of mice in the model group were significantly higher than those in the control group. Compared with the Sh-NC + model, the Sh-Cav1 + model group exhibited no significant difference in blood glucose, whereas the insulin level and HOMA-IR decreased significantly (Fig. 1C). Serum TG, TC, and LDL-C in the model group were all elevated, and those in the Sh-Cav1 + model group dropped compared with those in the Sh-NC + model group, whereas HDL-C was opposite (Fig. 1D).

### Cav1 on Endothelial Injury

**Fig. 2:**
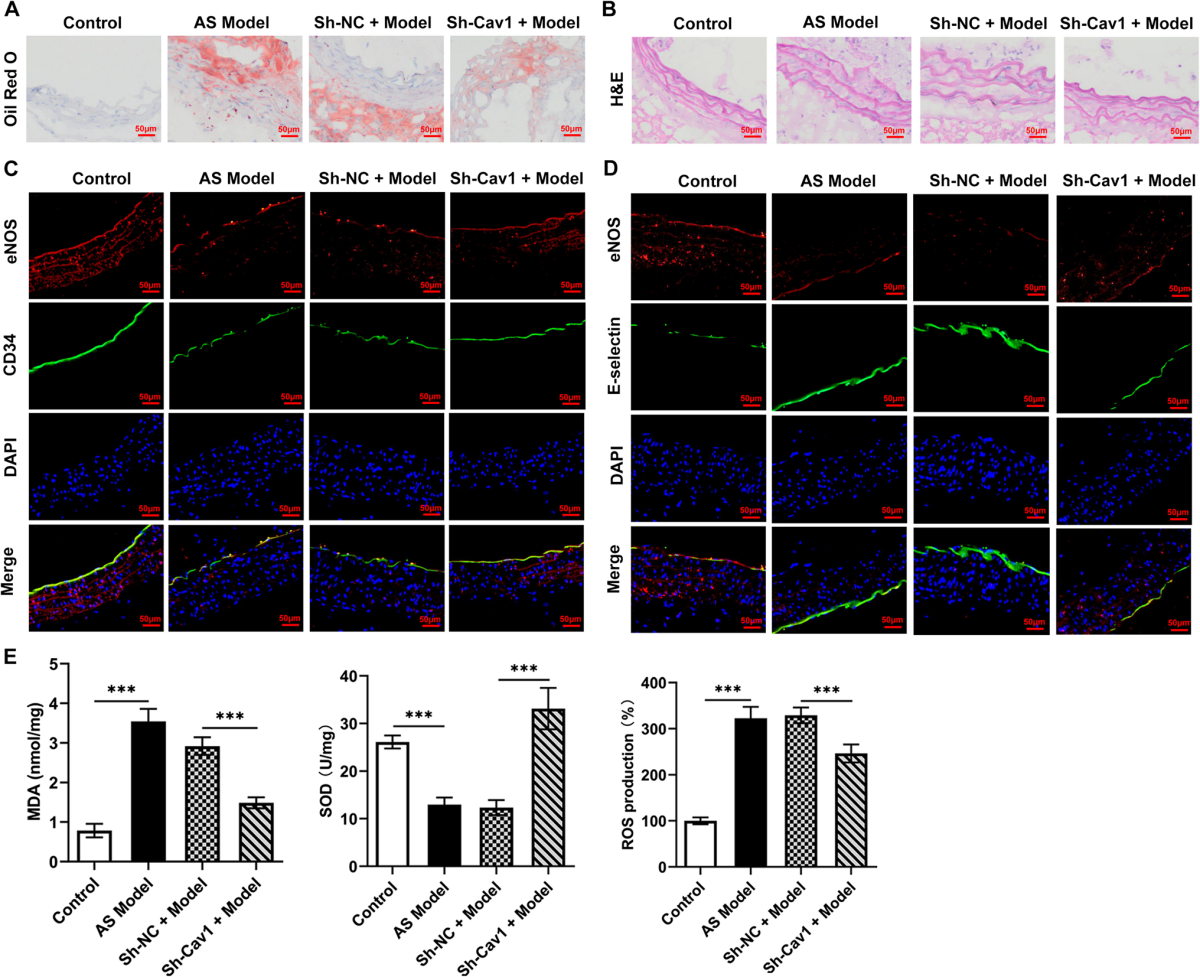
Cav1 on endothelial injury. **A** The degree of lipid deposition was revealed using Oil Red O staining. **B** Pathological damage of aorta was assessed using H&E staining. **C** The enrichment of eNOS in the aortic tissue was assessed using immunofluorescence, CD34 as an endothelial-specific marker, and DAPI to label nuclei. **D** The enrichment of E-selectin in the aortic tissue was assessed using immunofluorescence. **E** The levels of oxidative stress indicators were detected using kits. ^***^P < 0.001

Oil Red O staining revealed that there was obvious lipid deposition in the aorta of the AS group, and the degree of lipid deposition in the Sh-Cav1 + model group was weaker than that in the Sh-NC + model group (Fig. 2A). H&E staining showed that the aortic wall in the AS group was obviously thickened, and the arrangement of cells was disordered, and Sh-Cav1 could reduce such endothelial injury (Fig. 2B). IF demonstrated that AS induced a decrease in the eNOS in the aortic tissue, and Sh-Cav1 could alleviate this to some extent (Fig. 2C). Whereas, endothelial injury marker E-selectin increased in the model group, and reduced in response to Sh-Cav1 compared with the Sh-NC + model group (Fig. 2D). In addition, ROS and MDA increased and SOD decreased in the AS group tissues, and Sh-Cav1 could alleviate the alterations of these oxidative stress indicators (Fig. 2E).

### Signaling Pathways

**Fig. 3:**
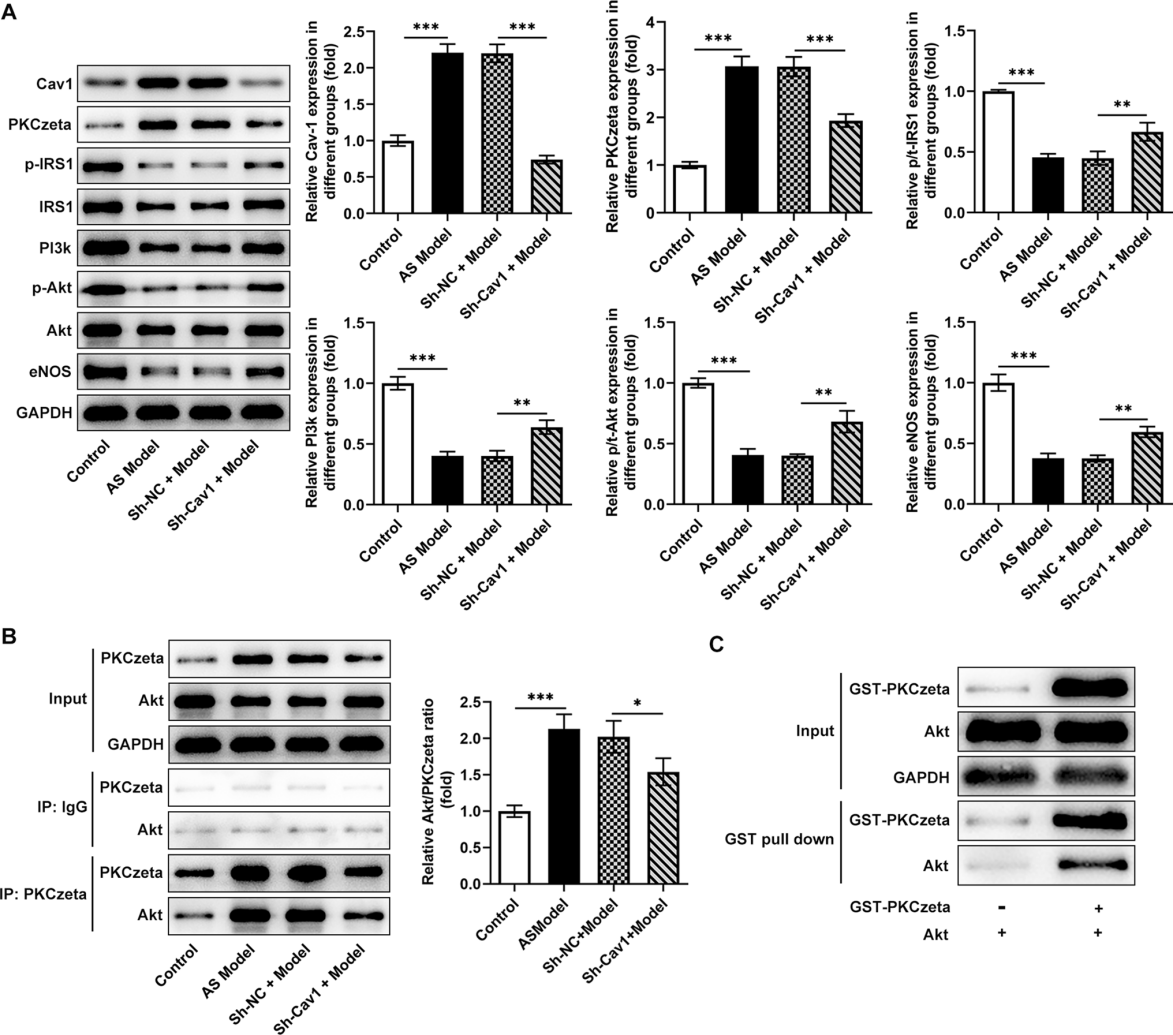
Two signaling pathways that regulate Akt. **A** The contents of Cav1, PKCzeta, and IRS1/PI3k/Akt/NOS pathway–related proteins in tissues were measured using western blotting. **B** Co-IP and **C** Pull-down experiments were applied to evaluate the binding between PKCzeta and Akt. ^*^P<0.05, ^**^P<0.01, ^***^P<0.001

The contents of Cav1, PKCzeta, and PI3k/Akt/NOS pathway–related proteins in tissues were measured using western blotting. Cav1 and PKCzeta increased in the AS group, while p/t-IRS1, PI3K, p/t-Akt, and eNOS decreased in the AS group. Sh-Cav1 could significantly hinder the changes in these protein levels, indicating that this pathway was blocked (Fig. 3A). The results of Co-IP and Pull-down experiments displayed that PKCzeta could bind to Akt, AS promoted the Akt/PKCzeta ratio, and Sh-Cav1 reduced the Akt/PKCzeta ratio, which might be due to the reduced PKCzeta failing to produce sufficient antagonistic effect on Akt (Fig. 3B, C).

### Validation of Functional Phenotypes

**Fig. 4:**
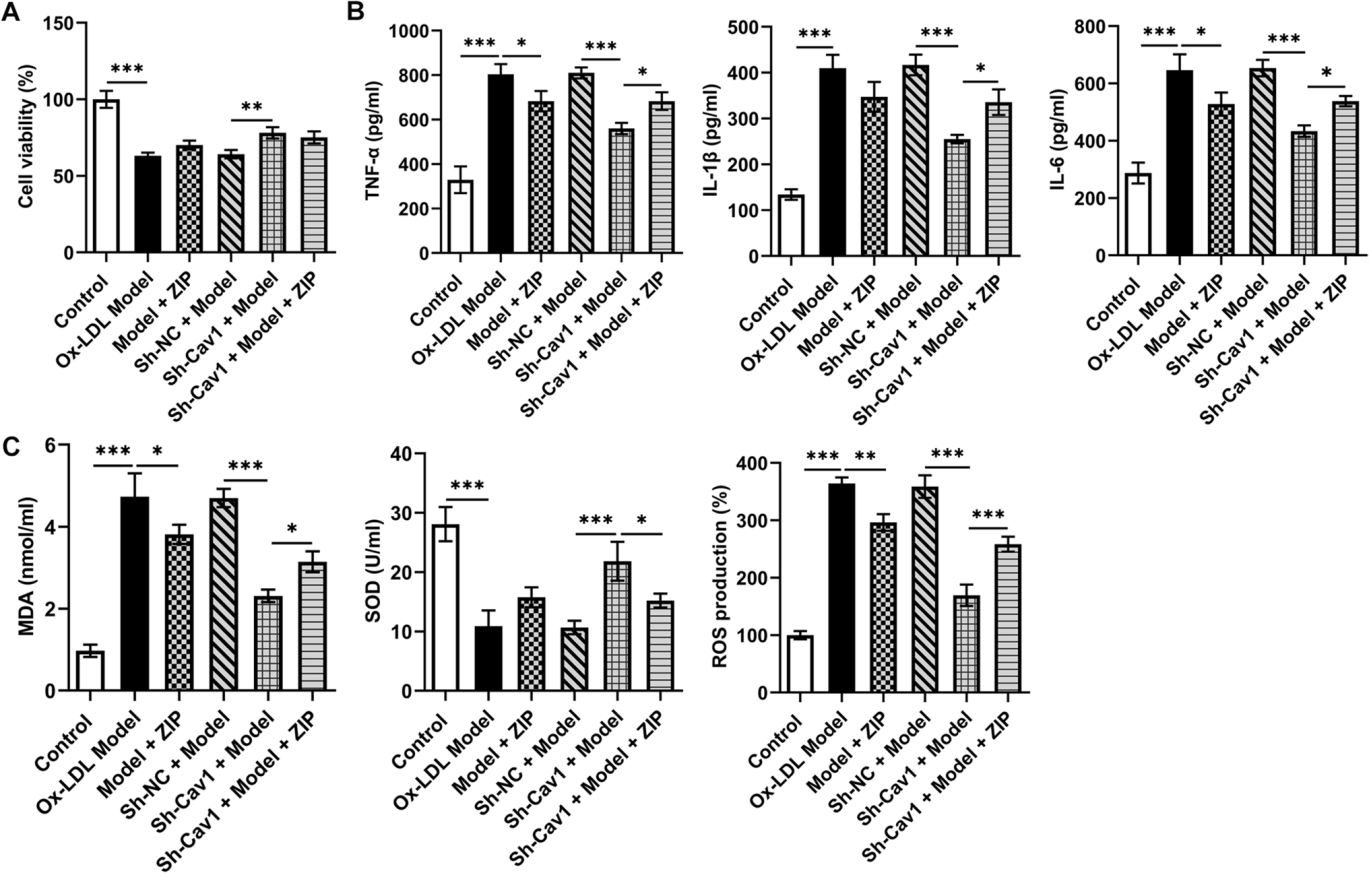
Cav1 and ZIP on cellular phenotypes. **A** The viability of HUVECs was measured using the CCK8 assay. **B** Inflammatory factors IL-6, IL-1β, and TNF-α in cell supernatants were measured. **C** The levels of oxidative stress in HUVECs were detected using kits. ^*^P<0.05, ^**^P<0.01, ^***^P<0.001

**Fig. 5:**
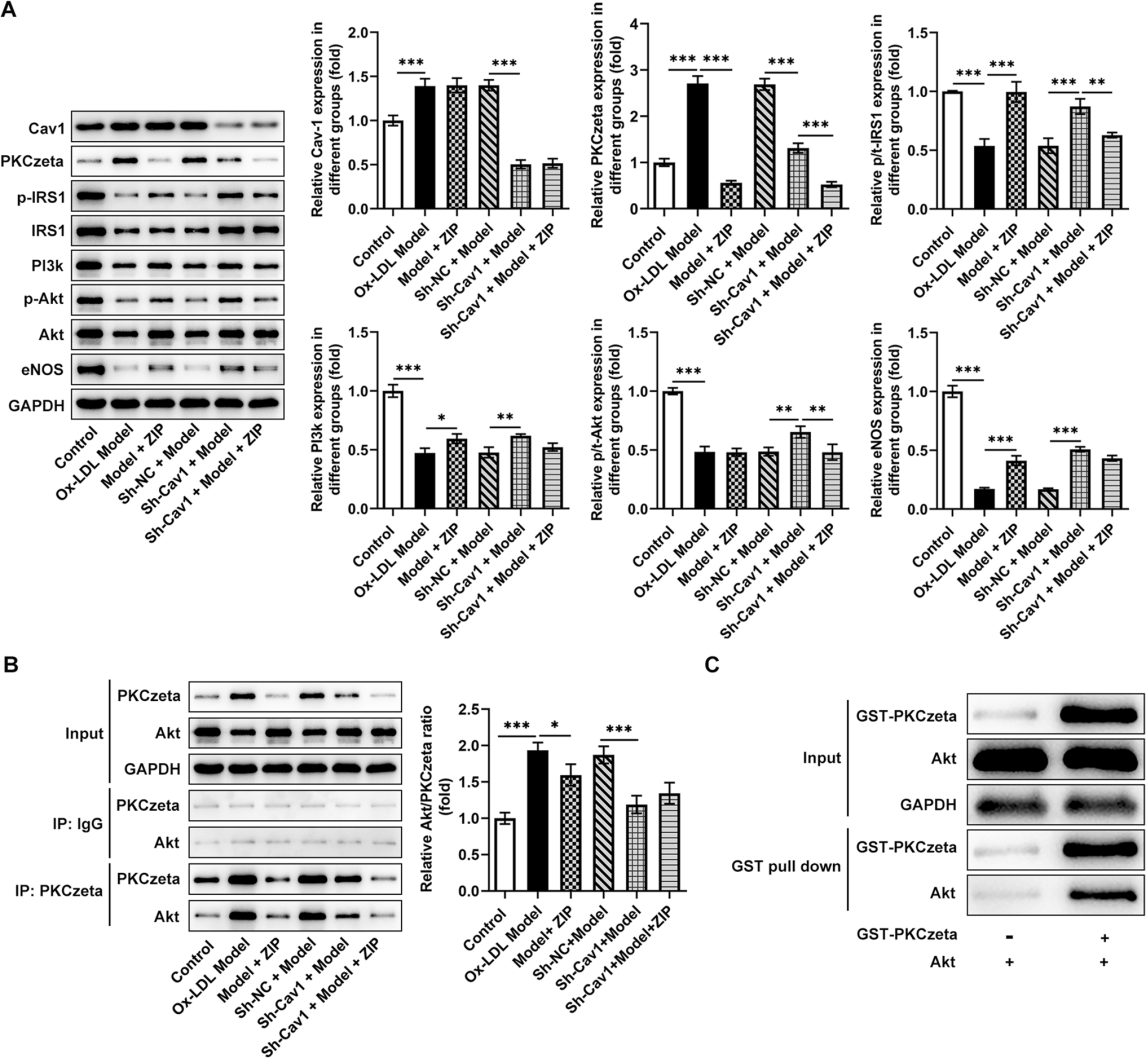
Cav1 and ZIP on signaling pathways. **A** The contents of Cav1, PKCzeta, and IRS1/PI3k/Akt/eNOS pathway–related proteins in HUVECs were measured using western blotting. **B** Co-IP and **C** Pull-down experiments were applied to evaluate the binding between PKCzeta and Akt in HUVECs. ^*^P<0.05, ^**^P<0.01, ^***^P<0.001

Treatment of ox-LDL induced HUVEC injury and gave ZIP treatment, and CCK8 assay revealed that ZIP increased the viability of untransfected cells; however, compared with the Sh-Cav1 + model group, ZIP decreased cell viability in the Sh-Cav1 + model + ZIP group (Fig. 4A). This was due to the fact that PKCzeta had a positive effect without being coupled by Cav1. Inflammatory factors IL-6, IL-1β, and TNF-α in cell supernatants were decreased by ZIP, and ZIP abolished the attenuation of inflammatory responses by Cav1 knockdown in HUVECs (Fig. 4B). Similarly, the degree of oxidative stress in HUVECs was attenuated by ZIP, which also abolished the suppression of oxidative stress by Cav1 knockdown (Fig. 4C). Western blot results revealed that ZIP did not significantly affect Cav1 enrichment, but significantly increased PI3k, p/t-IRS1, and eNOS protein enrichment. In Cav1-knockdown HUVECs, ZIP significantly decreased p/t-IRS1 and p/t-Akt ratios, and slightly decreased PI3k and eNOS protein enrichment (Fig. 5A). In addition, ZIP reduced Akt/PKCzeta ratio in the untransfected cells, but not significantly in sh-Cav1 knockdown cells (Fig. 5B, C). This indicated that ZIP itself did not affect the binding of PKCzeta to Akt under Cav-1/PKCzeta coupling.

## Discussion

The sharp rise in AS cases poses a great threat to human health worldwide, and the risk of developing AS in people with metabolic diseases has increased significantly over the past few decades [28, 29]. IR has been identified as a pivotal mediator between metabolic diseases and AS. A growing number of investigators have proposed that regulation of IR is even closer to the pathogenesis of AS than lipid disturbances [30]. Pathologically, the decline of β-cell function and insulin action induces hyposensitivity, which exacerbates lipid disorders, hyperglycemia, and abnormal fibrinolysis [31]. Abnormal adipocytes activate inflammatory responses by releasing pro-inflammatory factors, and this subclinical systemic inflammatory response not only mediates IR, but also participates in plaque rupture and thrombosis during AS [32, 33]. Meanwhile, the persistent inflammatory response induced by increased circulating triglycerides, free fatty acids, and cholesterol drives endothelial cell dysfunction, which further mediates alterations in insulin signaling pathways in muscle and liver tissue and disrupts glucose homeostasis [34].

An exogenous rise or an endogenous rise in blood glucose in response to abnormalities in cellular receptors such as GLUT4 that regulate glucose energy metabolism can lead to a feedback rise in insulin [35, 36]. Long-term high insulin leads to impairment of insulin signaling pathway at the level of IRS-1, resulting in decreased glucose transport/phosphorylation/metabolism, abnormal NO metabolism mechanism of vascular endothelial cells and smooth muscle cells, and inhibition of eNOS [37]. The down-regulation of eNOS leads to the reduction of NO biological activity, and its biological effects such as anti-infection, anti-oxidative stress, and inhibition of smooth muscle proliferation and migration are correspondingly weakened [38]; the reduction of NO bioavailability is accompanied by elevation in angiotensin II and free fatty acids, exacerbating levels of oxidative stress that can further worsen endothelial function [39–41]. In addition, the accumulated free radicals generated by lipid peroxidation in turn inhibit the bio-utilization of NO and promote the release of inflammatory factors and adhesion molecules [42]. This work delves further into the regulatory mechanisms that lead to IR, eNOS malfunction, and endothelial cell damage. That is, from the perspective of the IRS-1 signaling pathway, it displays the competitive antagonism between Cav1/PKCzeta and IRS1/PI3K. Their opposing effects on Akt activation destabilize NO production and are of note in the development of therapeutics that rely on regulatory mechanisms. Nevertheless, further study is required before this insight may be converted into outcomes.

Although existing therapy choices are effective for AS and some of its consequences, novel therapeutic techniques are still desperately needed. Drug design and kinetic simulations targeting Cav-1 appear to be a potentially fruitful strategy. Going forward, drugs acting on endothelial cells in AS-prone areas to reprogram the vasoprotective phenotype could counteract the effects of systemic risk factors (e.g., hypercholesterolemia). To sum up, Cav-1/PKCzeta influences insulin signaling and is directly linked to intrinsic vessel wall processes, emphasizing potential prospects for the development of selective therapeutics for endothelial dysfunction in the development of AS.

## Data Availability

The datasets used and/or analyzed during the current study are available from the corresponding author on reasonable request.
